# Chewing efficiency and patient-centered outcomes in maxillary rehabilitation with zygomatic vs. conventional implant-supported fixed restorations

**DOI:** 10.3389/froh.2026.1691698

**Published:** 2026-02-06

**Authors:** Lijuan Ye, Jie Li, Yuwei Dai, Xiaowan Ling, Lijun Yan, Feng Wang, Yiqun Wu

**Affiliations:** 1 Department of Second Dental Center, Shanghai Ninth People’s Hospital, Shanghai Jiao Tong University School of Medicine, College of Stomatology, Shanghai Jiao Tong University, Shanghai, China; 2 National Center for Stomatology, National Clinical Research Center for Oral Diseases, Shanghai Key Laboratory of Stomatology, Shanghai Research Institute of Stomatology, Research Unit of Oral and Maxillofacial Regenerative Medicine, Chinese Academy of Medical Sciences, Shanghai, China; 3 Department of Stomatology, Xuzhou Central Hospital Affiliated to Southeast University, Xuzhou, China; 4 Division of Oral and Maxillofacial Surgery, Faculty of Dentistry, Prince Philip Dental Hospital, The University of Hong Kong, Hong Kong, Hong Kong SAR, China

**Keywords:** chewing efficiency, conventional implants, edentulous maxilla, oral health-related quality of life, zygomatic implants

## Abstract

**Purpose:**

This study aimed to assess whether zygomatic implant-supported fixed restorations (ZIFRs) provide comparable chewing efficiency and patient-centered outcomes to conventional implant-supported fixed restorations (CIFRs) in edentulous maxilla cases.

**Material and methods:**

A total of 29 patients were enrolled in this study. These patients with maxillary edentulism received ZIFR (16) or CIFR (13) treatment between October 2018 and April 2022. Chewing efficiency was evaluated by the subjective assessments of the scales of chewed gums and the variance of the Hue in a two-color chewing gum mixing ability test. In addition, the Oral Health Impact Profile-14 (OHIP-14) was employed to compare the oral health-related quality of life of patients between these two groups.

**Results:**

There were no significant differences in chewing efficiency between the ZIFR and CIFR groups according to the results of the subjective assessments (*P* = 0.59 and *P* = 0.19 in the first and second stroke of the chewing test, respectively) and the variance of the Hue (*P* = 0.55 in the first stroke and *P* = 0.28 in the second stroke). Based on OHIP-14 results, the ZIFR group reported less psychological pain, physical disability, and psychological discomfort. In addition, the success rate of implants in the ZIFR and CIFR groups was 100% and 98.1% at the implant level and 100% and 93.75% at the patient level, respectively.

**Conclusions:**

With the limitations of the study, the chewing efficiency of ZIFRs was comparable to CIFRs in the rehabilitation of maxillary edentulism. However, under such conditions, ZIFRs can offer better postoperative quality of life compared to CIFRs.

## Introduction

Dental implants have been widely applied to repair dentition defects due to their considerable long-term success rates (1). Nevertheless, this strategy faces challenges in edentulous cases with severely atrophic maxillae, owing to inadequate bone volume surrounding dental implants (2). To overcome this issue, various approaches, such as maxillary sinus floor elevation, short implants, pterygoid implants, and zygomatic implants (3–7), have been proposed for dental implant-supported rehabilitations.

Among these approaches, zygomatic implants have gained increasing attention because of their high success rate and predictability in extremely atrophic maxillae (8). A systematic review documented a 12-year cumulative success rate of 95.21% across 4,556 zygomatic implants in 2,161 patients (9). However, some concerns remain regarding the complications of zygomatic implants, for example, maxillary sinusitis, infra-orbital paresthesia, oroantral fistula, and orbital perforations (10, 11). Although most studies have focused on these severe complications, chewing efficiency and patient-centered outcomes—the two main parameters for evaluating the efficacy of implant therapy after zygomatic implant treatment—have rarely been investigated.

Some characteristics of zygomatic implants may influence chewing efficiency and patient-centered outcomes. For potentially decreased chewing efficiency, zygomatic implant-supported fixed restorations (ZIFRs) involve short-span arch designs. Thus, the prostheses may even eliminate one or two premolars due to an aberrant form of the maxillary arch in patients with severely atrophic maxillae. In addition, ZIFRs frequently require a long cantilever in anterior areas to tackle complicated soft and hard tissue defects. ZIFRs may also lead to compromised postoperative quality of life for patients—for instance, by narrowing the space for tongue movement.

Whether these characteristics will negatively affect the chewing and postoperative subjective outcomes of patients remains unclear. Therefore, this study was designed to distinguish the chewing efficiency and patient-centered outcomes between ZIFRs and conventional implant-supported fixed restorations (CIFRs) in edentulous maxilla patients. The results of this study indicate that while chewing efficiency of ZIFRs is comparable with CIFRs, the patient-centered outcomes of ZIFRs are superior.

## Materials and methods

### Ethical approval

This study complied with the requirements of the Declaration of Helsinki requirements and was approved by the local ethics committee at Shanghai Ninth People's Hospital, Shanghai Jiao Tong University of Medicine, China (SH9H-2022-T25-1). All patients provided written informed consent.

### Patient enrollment

A total of 29 edentulous maxilla patients were enrolled in this study according to the inclusion and exclusion criteria. These patients received either ZIFR or CIFR treatment in the Department of Second Dental Center, Shanghai Ninth People's Hospital, between October 2018 and April 2022. The inclusion criteria were as follows: (i) patients ≥18 years who provided written informed consent; (ii) edentulous maxilla patients [Cawood and Howell classification V and VI (12)] who had received ZIFR or CIFR treatment, including one-piece restorations and segmental bridges; (iii) prosthesis delivery completed more than one year prior to enrollment; and (iv) patients with intact mandibular dentition consisting of natural teeth, implant-supported restorations, or a combination of both. The exclusion criteria were as follows: (i) suffering due to uncontrolled systemic and/or mental diseases; (ii) impaired mandibular dentition; (iii) mandibular removable (partial/full) dentures and/or implant-supported overdentures; and (iv) malocclusal deformities and aberrant occlusal relationships.

### Implant placement and success rate analysis

**Figure 1:**
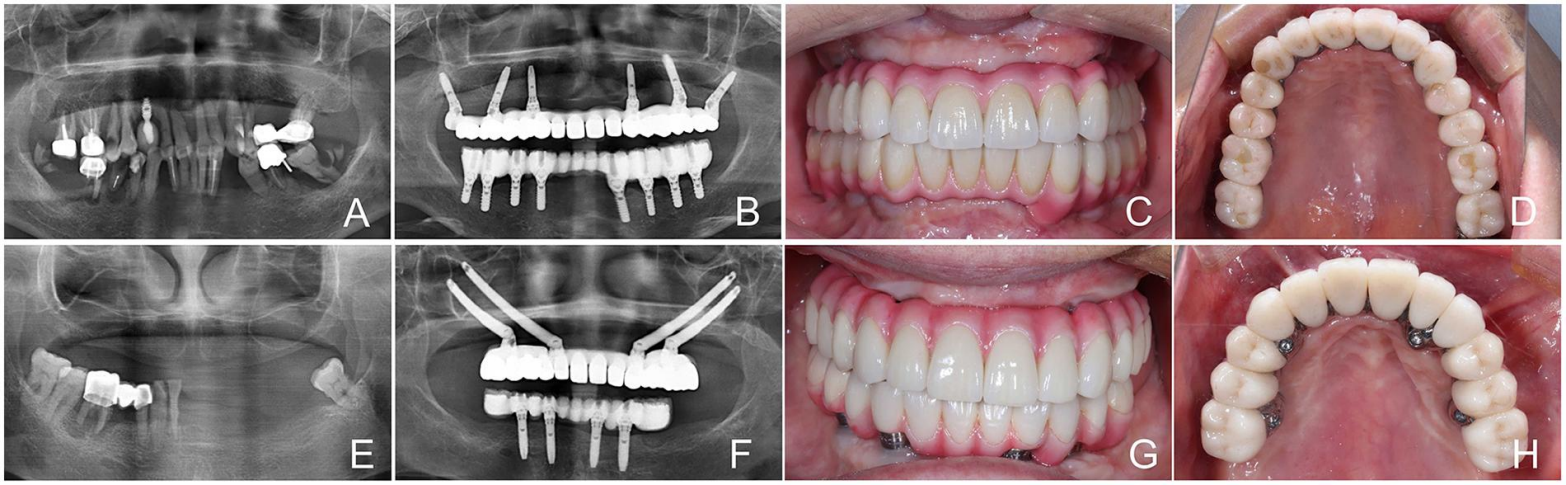
Representative cases of CIFR **(A–D)** and ZIFR **(E–H)**. **(A)** Preoperative panoramic radiograph of a CIFR case; **(B)** panoramic radiograph after prosthesis delivery; **(C)** intraoral photograph; **(D)** intraoral photographs of the occlusal surface; **(E)** preoperative panoramic radiograph of a ZIFR case; **(F)** panoramic radiograph after prosthesis delivery; **(G)** intraoral photograph; and **(H)** intraoral photographs of the occlusal surface.

All implant placement procedures were performed by a senior specialist in dental implantology, Y. W., with the assistance of F. W. (Figure 1). In the ZIFR group, the quad zygoma protocol was applied that involved the following: Four zygomatic implants were bilaterally placed, or alternatively, a single zygomatic implant on each side combined with 2–4 conventional dental implants was applied, depending on the bone defect in the posterior areas of the maxillae (13). Success rates were evaluated at both the implant and patient levels, by the percentage of functional prostheses at the end of the last follow-up. Furthermore, mechanical complications, such as chipping, fracture of restorations, or loosening of abutment screws, were also recorded.

### Chewing efficiency assessment

**Figure 2:**
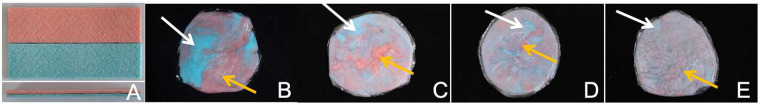
Chewing gums and SAs. **(A)** Chewing strip consists of two gums with different colors; **(B)** SA2, most of chewing gum unmixed; **(C)** SA3, bolus slightly mixed, but bits of unmixed original color; **(D)** SA4, bolus well mixed, but color not uniform; and **(E)** SA5, bolus perfectly mixed with uniform color. White arrow: blue gum; yellow arrow: orange gum.

**Figure 3:**
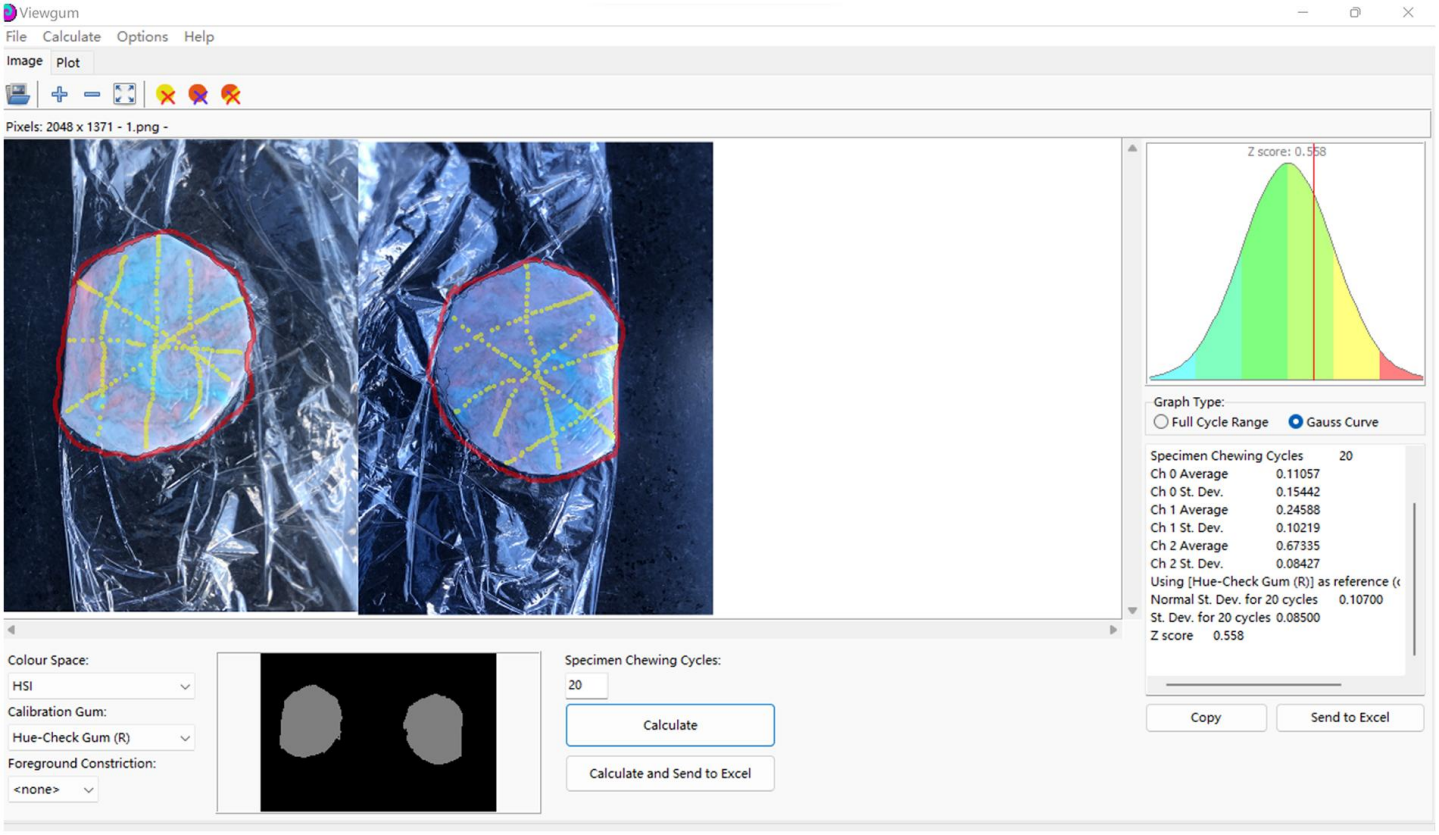
The Hue of images of chewed gum analyzed using Viewgum.

Chewing efficiency was evaluated using a two-color chewing gum mixing ability test (14). Each patient received a chewing gum strip consisting of blue and red gums (Mars Wrigley Confectionery, Guangzhou, China) (Figure 2). Patients were instructed to chew for 20 cycles, equally distributed between the left and right sides (stroke 1). After a 3-min rest, a second chewing cycle (stroke 2) was performed. The chewing cycles were counted by the investigators and patients. After chewing, the gum was collected in a transparent plastic pouch, avoiding any saliva accumulation within the pouch. Afterward, the collected chewing gums were flattened to a uniform 1 mm thickness for sequential scanning and visual analysis by Y. D. using Viewgum (http://www.dhal.com) (15) (Figure 3). Chewing efficiency was quantified based on the variance of the Hue (VOH) within the image (14). In addition, subjective assessments (SAs) of how much gum was mixed were performed by L. Y. and categorized into five scales: SA1, the chewing gum strip was unmixed, had cusp impressions, or was folded once; SA2, large chunks of unmixed chewing gum remained; SA3, bolus was partially mixed but contained traces of the original color that had not been mixed; SA4, well-combined bolus but not uniform in color; and SA5, uniform color with perfect mixing of the bolus.

### Patient-centered outcomes: OHRQoL assessed by OHIP-14 questionnaire

The Oral Health Impact Profile-14 (OHIP-14) was used to evaluate oral health-related quality of life (OHRQoL). The questionnaire assessed functional limitation, pain, physical and psychological incapacity, psychological discomfort, handicap, and social disability. Participants rated the frequency of these items utilizing a 5-point Likert scale: 0, 1, 2, 3, and 4 referring to “never,” “hardly ever,” “occasionally,” “fairly often,” and “very often,” respectively.

### Statistical analysis

The statistical analyses included descriptive statistics and inferential statistics. Descriptive statistics—including means, medians, SD, minimum and maximum values, and 95% confidence intervals (CI)—were used to characterize the SA, VOH, and OHIP. A Mann–Whitney *U* test compared the VOH, SA, and OHIP values. In addition, cumulative implant survival rates were calculated and expressed as percentages. Statistical analyses were performed using SPSS Statistics 26 (IBM, Armonk, NY, USA), with significance set at *P* < 0.05.

## Results

### Demography information

A total of 29 patients were recruited for this study. The details of patient characteristics are summarized in Table 1. The ratio of patients with natural dentition, implant-supported fixed restorations, and a mix of the two in the mandible was 4:5:4 in the ZIFR group and 1:9:6 in the CIFR group. According to the Cawood and Howell classification, the distribution of patients of Classes V and VI in the ZIFR and CIFR groups was 6:7 and 9:7, respectively, demonstrating a comparable baseline of the atrophy of maxillae.

**Table 1:** Patients in the ZIFR group.

Patients	Age (years)	Gender	Cawood and Howell classification	Smoking	History of systematic disease	Opposing dentition	Immediate loading	No. of implants with BOP+	No. of crowns	Implant numbers (position)		
										Zygomatic implants	Conventional implants	Failed
1	45	Female	VI	No	No	D	Yes	0	12	4 (16, 13, 23, 26)	0	0
2	56	Female	VI	No	Diabetic, Cardiovascular disease	N, D	Yes	0	12	4 (15, 12, 22, 25)	0	0
3	50	Male	VI	Yes	Diabetic, Cardiovascular disease	N, D	Yes	0	13	4 (15, 13, 33, 35)	2 (11, 21)	0
4	46	Male	V	No	Hypertension	D	Yes	0	12	2 (15, 25)	4 (14, 12, 21, 23)	0
5	52	Male	V	No	No	N	Yes	0	12	2 (16, 26)	4 (14, 11, 24, 21)	0
6	65	Female	VI	No	No	N	NO	0	12	4 (16, 13, 23, 26)	0	0
7	62	Male	VI	Yes	Hypertension	N	Yes	4	14	4 (17, 15, 25, 27)	0	0
8	33	Female	VI	No	No	D	Yes	0	12	4 (16, 13, 23, 26)	2 (11, 21)	0
9	43	Male	V	No	Hypertension	D	Yes	0	13	4 (16, 13, 23, 26)	0	0
10	47	Male	V	No	Depression	N, D	Yes	0	12	2 (16, 26)	4 (13,11,21,23)	0
11	47	Female	V	No	No	D	Yes	6	12	2 (16, 26)	4 (13, 11, 21, 23)	0
12	45	Female	V	No	No	N	Yes	4	12	2 (16, 26)	2 (13, 23)	0
13	59	Female	VI	No	No	N, D	Yes	6	12	2 (16, 26)	4 (13, 11, 21, 23)	0

D, dental implant-supported prostheses; N, natural teeth; BOP, bleeding on probing.

### Survival and success rates of implants

All patients underwent follow-up 25.8 ± 3.6 months (mean ± SD, range: 12–48 months). In the ZIFR group, seven patients received the quad approach, while the others received bilaterally single ZIFR with 2–4 conventional dental implants (Table 1). The implant success rate in the ZIFR group was 100% at both the implant and patient levels (66/66 implants and 13/13 patients). In the CIFR group, 13 patients received 105 conventional dental implants (Table 2), and the success rate in the group was 98.1% at the implant level (103/105 implants) and 93.75% at the patient level (15/16 patients).

**Table 2:** Patients in the CIFR group.

Patients	Age (years)	Gender	Cawood and Howell classification	Smoking	History of systemic disease	Opposing dentition	Immediate loading	No. of implants with BoP+	No. of crowns	Implant numbers (position)	Failed
1	67	Female	VI	Yes	Diabetes	N	Yes	6	12	6 (16, 14, 12, 22, 24, 26)	0
2	63	Female	VI	No	Hypertension	D	Yes	0	14	6 (17, 14, 12, 22, 24, 27)	0
3	62	Male	VI	Yes	Hypertension	N	Yes	1	12	7 (17, 15, 13, 11, 23, 25, 27)	0
4	47	Male	V	No	Diabetes	D	Yes	0	12	6 (16, 14, 12, 21, 24, 26)	0
5	74	Female	VI	No	Hypertension, Diabetes	D	Yes	3	12	7 (17, 16, 14, 13, 23, 25, 27)	2
6	57	Female	V	No	Cardiovascular disease	N	No	0	12	6 (16, 14, 12, 22, 24, 26)	0
7	50	Male	V	Yes	Hypertension	D	No	2	14	7 (16, 15, 12, 21, 22, 26, 27)	0
8	40	Female	V	No	No	N	Yes	0	13	8 (17, 15, 13, 11, 21, 23, 25, 27)	0
9	56	Male	VI	Yes	No	N, D	Yes	0	12	8 (17, 15, 13, 11, 21, 23, 25, 27)	0
10	55	Male	V	Yes	Hypertension	D	Yes	0	13	6 (13, 11, 21, 23, 25, 27)	0
11	59	Female	VI	Yes	No	N, D	Yes	1	12	6 (16, 14, 12, 22, 24, 26)	0
12	50	Male	V	Yes	No	D	Yes	0	12	6 (16, 14, 12, 22, 24, 26)	0
13	58	Male	V	No	No	N, D	Yes	0	12	6 (16, 14, 12, 22, 24, 26)	0
14	40	Female	VI	No	No	D	Yes	0	12	6 (17, 15, 12, 22, 25, 27)	0
15	58	Male	V	Yes	No	D	No	0	12	6 (16, 14, 12, 22, 24, 26)	0
16	65	Male	V	No	No	D	Yes	0	14	8 (17, 15, 14, 12, 22, 24, 26, 27)	0

D, dental implant-supported prostheses; N, natural teeth; BOP, bleeding on probing.

### Rehabilitation strategy

After taking into consideration patient preference and budget, in the ZIFR group, five patients obtained a titanium framework and all-ceramic crowns; six individuals received a titanium framework and polymeric porcelain resin; and two received a titanium framework and acrylic resin prosthetic teeth. In the CIFR group, 11 participants received a one-piece titanium framework and all-ceramic crowns; four had a titanium framework alongside polymeric porcelain resin; and one participant had a titanium framework as well as acrylic resin prosthetic teeth.

### Complications

Two patients in the CIFR group experienced chipping after 1 year of follow-up. Two patients in both the ZIFR group (2/13) and the CIFR group (2/16) complained about bruxism after prosthesis delivery, after which a night guard was provided.

### Chewing efficiency assessment

Images of chewed gums were collected (Figure 3), and SAs and VOH values are provided in Table 3. Although the SA scores and VOH values of the CIFR group were higher, there was no significant difference after stroke 1 and stroke 2 chewing.

**Table 3:** SA and VOH in the ZIFR and CIFR groups.

Strokes	SA			VOH		
	ZIFR	CIFR	*P*-value	ZIFR	CIFR	*P*-value
First stroke						
Mean/	/	/	0.59	0.401	0.456	0.55
95% CI				[0.294; 0.525]	[0.348; 0.557]	
Median	4	3		0.383	0.464	
SD	/	/		0.214	0.203	
Min	3	2		0.064	0.136	
Max	4	5		0.813	0.794	
Second stroke						
Mean/	/	/	0.19	0.358	0.477	0.28
95% CI				[0.239; 0.503]	[0.365; 0.592]	
Median	4	3		0.322	0.495	
SD	/	/		0.233	0.214	
Min	3	2		0.064	0.174	
Max	5	5		0.794	0.843	

### OHRQoL assessed by the OHIP-14 questionnaire

Following prosthesis delivery, all patients completed the OHIP-14 questionnaire. The ZIFR group demonstrated lower OHIP-14 scores than the CIFR group. Table 3 presents the seven dimensions of the OHIP-14 and related OHRQoL variables. With regard to the dimension of physical pain, the ZIFR group scored 0.62, while the CIFR group scored 0.75. Similarly, on the dimension of psychological discomfort and disability, the ZIFR group scored less than the CI group, which indicates that the zygomatic technique exerted less negative effect on patients' social life (Table 4).

**Table 4:** OHIP-14 score in the ZIFR and CIFR groups.

Dimensions	ZIFR		CIFR		*P*-value	Difference between ZIFR and CIFR	
	Mean/ 95% CI	SD	Mean/ 95% CI	SD		Mean/ 95% CI	SD
Functional limitations	1	0.82	0.94	1	0.41	−0.135	0.34
	[0.65; 1.35]		[0.56; 1.34]			[−0.706, 0.436]	
Physical pain	0.62	0.64	0.75	1.04	0.01	−0.24	0.32
	[0.35; 0.88]		[0.34; 1.22]			[−0.798, 0.318]	
Psychological discomfort	0.77	0.63	0.94	1.33	0.01	−0.192	0.38
	[0.46; 1.08]		[0.50; 1.47]			[−0.751, 0.367]	
Physical disability	0.62	0.71	0.78	1.1	0.37	−0.067	0.34
	[0.31; 0.92]		[0.41; 1.22]			[−0.637, 0.503]	
Psychological disability	0.65	0.72	0.84	1.38	0.04	−0.192	0.4
	[0.38; 0.96]		[0.38; 1.38]			[−0.751, 0.367]	
Social disability	0.27	0.48	0.69	1.04	0.34	−0.096	0.29
	[0.08; 0.50]		[0.31; 1.16]			[−0.655, 0.463]	
Handicap	0.35	0.47	0.88	1.13	0.06	−0.192	0.31
	[0.15; 0.58]		[0.44; 1.34]			[−0.751, 0.367]	
Global	4.28	3.63	5.82	7.66	<0.01	−0.159	2.16
	[1.96; 6.58]		[1.98; 9.27]			[−5.72; 3.01]	

## Discussion

To our knowledge, this present study is the first one to explore chewing efficiency and postoperative quality of life of patients who have undergone ZIFR treatment for edentulous maxillae. Our findings suggest that the chewing efficiency of ZIFRs is comparable with that of CFRIs. In addition, ZIFRs present better postoperative quality of life for patients than CIFRs. Both ZIFR and CIFR strategies achieved considerable success rates for implants, with no severe complications identified. Taken together, ZIFRs represent an approach for reconstructing thoroughly impaired maxillary dentition, particularly in cases with extremely severe posterior bone defects.

The promising success rate of zygomatic implants has been well studied. A multicenter randomized clinical trial reported that zygomatic implants with immediate loading led to a significant increase in both prosthetic and implant success compared with sinus augmentation and delayed implant positioning after 1 year of follow-up (16). Similarly, Sales et al. reported a 96.7% success rate of ZIFRs after reviewing seven systematic reviews involving 2,313 patients with 4,812 zygomatic implants (17). In the present study, ZIFRs achieved a success rate consistent with a 17-year retrospective follow-up analysis. Although two failed implants occurred in the CIFR group (18), both were in a female patient—the oldest age among the 29 patients—who had two systemic diseases (hypertension and diabetes mellitus). These conditions could disturb normal osseointegration. The failure emphasizes the challenges and requirement for comprehensive evaluation when offering dental implant treatment to patients.

Based on the classification of atrophic maxillae, both the quad zygoma approach and single zygomatic implant with conversational implants were administered in the present study. The quad zygoma approach can reduce cantilever length and confer prosthetic stability, even allowing immediate prosthetic loading in severely atrophic edentulous maxillae (19). In this study, seven patients received quad zygoma implants due to severely resorbed maxillae. The chewing efficiency and postoperative reactions of these patients were not compared due to complicated influencing factors.

While ZIFRs demonstrated appealing clinical outcomes, their indications are restricted, as mentioned in abundant studies (20, 21). Owing to the long path that is adjacent to vital anatomic structures, it is imperative to manage and address mechanical and biological complications, such as pain, bruise, and recurrent maxillary sinusitis (22, 23). In the present study, various alternatives to ZIFR treatment were offered to patients, including lateral window sinus floor evaluation, onlay bone grafting, split bone block technique, and guided bone regeneration. In line with concerns regarding prolonged healing, unpredictable outcomes, and social needs, some patients opted for ZIFRs with immediate loading. Meanwhile, in some of the complicated ZIFR cases, dynamic navigation was employed to reduce surgical risks and achieve significant rehabilitation outcomes. No severe complications were observed during follow-up, and a prolonged evaluation is ongoing.

Although chewing gum has been widely used in evaluating chewing efficiency of implant-supported overdentures (24), there have been no studies comparing the efficiency of ZIFRs and CIFRs. Chewing gum is a time-saving, straightforward, and economical approach, enabling its application in geriatric wards or private practices in the absence of specific equipment or trained staff. In addition, gum has an elastic consistency, facilitating maximum muscle activation.

Although many questionnaires have been used to assess the quality of life, not all are suitable for evaluating OHRQoL. Among these questionnaires, OHIP is the prevailing one in OHRQoL evaluation (25). Originally proposed by Slade and Spencer in 1994 (26), OHIP aims to provide comprehensive data on patient attitudes regarding influence on their wellness. However, not all studies employ the full suite of 49 questions. Compared to the full OHIP, OHIP-14 has demonstrated excellent consistency, accuracy, and precision, making it more practical in clinical practice (27). Therefore, OHIP-14 has become widespread in dental research. In this study, the OHIP-14 scores of patients revealed a statistical difference between these two groups. In terms of physical pain, the ZIFR group scored lower than the CIFR group, which may be attributed to the use of general anesthesia to some extent. Similarly, on the dimension of psychological discomfort and disability, the ZIFR group performed better than the CIFR group, indicating that ZIFRs exert less negative impact on patients’ social life. However, the observed differences may not reflect authentic clinical discrepancies. A change of ≥3 points in OHIP-14 is generally considered meaningful (28); however, in this study, the change was less than 1, which may be attributed to the small sample size.

All procedures and analyses were conducted under realistic conditions in this study, providing clinical and instructive insights for future research. The data indicate that chewing efficiency between ZIFRs and CIFRs is comparable. In addition, postoperative quality of life is better in the ZIFR group. Nonetheless, caution should be exercised while interpreting outcomes due to the limited sample size. To generate stronger evidence, prolonged follow-ups and randomized controlled trials should be scheduled.

## Conclusions

Preliminary data reveal that the chewing efficiency of ZIFRs does not statistically differ from CIFRs. Moreover, ZIFRs are associated with higher patient satisfaction in the rehabilitation of edentulous maxillae compared with CIFRS. These findings suggest that ZIFRS represent a promising alternative to CIFRs in certain conditions.

## Data Availability

The original contributions presented in the study are included in the article/Supplementary Material, further inquiries can be directed to the corresponding authors.
